# The Effect of Direct Oral Anticoagulants on Gastric Mucosa and Helicobacter Pylori Prevalence in Dyspeptic Patients: A Retrospective Cross-Sectional Study

**DOI:** 10.7759/cureus.46477

**Published:** 2023-10-04

**Authors:** Kubilay Issever, Ersin Kuloglu, Ali Muhtaroglu, Demet Seker, Osman Kotur, Ahmet Cumhur Dulger

**Affiliations:** 1 Internal Medicine, Giresun University Faculty of Medicine, Giresun, TUR; 2 General Surgery, Giresun University Faculty of Medicine, Giresun, TUR; 3 Neurology, Imperial Hospital, Trabzon, TUR; 4 Gastroenterology, Giresun University Faculty of Medicine, Giresun, TUR

**Keywords:** intestinal metaplasia, dyspepsia, helicobacter pylori, gastric mucosa, direct oral anticoagulants

## Abstract

Background and aim

Although direct oral anticoagulants (DOACs) are widely used and their side effects related to bleeding at various body sites have been well studied in the literature, less is known about their local impact on gastric mucosa. Some studies suggest that the higher risk of gastrointestinal (GI) bleeding associated with DOACs may be due to their direct local anticoagulant effects on the gastric mucosa. In this study, we aim to evaluate whether this potential local effect has a favorable outcome on the gastric mucosa and the prevalence of *Helicobacter pylori* (HP).

Materials and methods

A total of 125 patients with dyspepsia were included in the study. Sixty patients who had been using a DOAC for at least one month were classified as the "DOAC group," while 65 patients who had not used DOACs were designated as the "control group." Demographic, laboratory, and pathological findings for these patients were retrospectively analyzed from their medical files.

Results

Patients in the DOAC group were significantly less likely to have antral gastritis (AnG) (p = 0.028), while the frequencies of HP and atrophic gastritis (AtG) were similar between the two groups. Although not statistically significant, the DOAC group showed fewer instances of intestinal metaplasia (IM) and a higher number of upper GI ulcers. Patients who had been using DOACs for more than 12 months had increased incidences of IM, upper GI ulcers, AnG, and HP compared to those who had been using DOACs for 12 months or less. The Rivaroxaban subgroup showed significantly lower HP positivity compared to patients using other DOACs (p = 0.042). Among all subgroups, the Rivaroxaban group had the lowest frequency of AnG (p = 0.024).

Conclusion

While DOACs seem to prevent AnG, HP, and IM at their early use stages, unfavorable gastric mucosa manifestations might increase with prolonged use. Higher upper GI ulcer prevalence is another controversial result of this issue. Rivaroxaban shines amongst other DOACs with its lesser HP and AnG association. These exciting findings should be supported by randomized controlled trials with large patient populations.

## Introduction

New oral anticoagulants or direct oral anticoagulants (DOACs) are a group of drugs that inhibit thrombin or factor Xa and are increasingly preferred over warfarin for their high safety, efficacy, and low side effect profile [1,2]. This drug group, consisting of dabigatran, rivaroxaban, apixaban, and edoxaban, has the advantage of being used in fixed doses without requiring any laboratory follow-up [3]. Due to these advantages, they have become the first choice for ischemic stroke prophylaxis in patients with venous thromboembolism and atrial fibrillation [4]. The incidence of GI tract bleeding in patients using these drugs is 1.5-2% per year, and the mortality rates in such cases can be as high as 16.2% [3]. In another study, delayed gastric bleeding complication was observed in 14% of patients using DOACs (18% for those using warfarin), who underwent endoscopic submucosal dissection. This rate was even significantly lower in patients using dabigatran (8%) compared to those using other DOACs and warfarin [5].

Studies conducted on patients using DOACs have shown that this drug group has a lower bleeding risk than warfarin in all other body systems, except for a higher bleeding risk specifically in the GI system [6]. The only hypothesis for the potential reason behind this was proposed in a 2013 study. While warfarin is rapidly absorbed from the gastric mucosa with a bioavailability of over 95% and therefore exhibits systemic effects, all DOACs, especially dabigatran, continue to be absorbed throughout the GI system due to their low bioavailability and can even be detected in feces. This characteristic of DOACs is thought to increase the risk of bleeding by exerting a direct local anticoagulant effect on any mucosal or submucosal lesions in the GI system [7]. A review of the literature reveals that although the side effects of this drug group on GI bleeding have been extensively studied, other potential impacts on the gastric mucosa are yet to be examined. Our hypothesis in this context is that DOACs may have a positive effect on gastric mucosal atrophy, metaplasia, and HP gastritis by increasing local blood flow in the gastric mucosa. Hence, in our study, we compared the laboratory and gastric specimen findings of dyspeptic patients who were either using or not using DOACs to assess whether DOACs have any influence on these parameters.

## Materials and methods

A total of 125 patients who visited the internal medicine and gastroenterology outpatient clinics at Giresun University Training and Research Hospital between 2020 and 2023 with complaints of dyspepsia were included in the study. The inclusion criteria were being over 18 years of age, having dyspepsia as the primary symptom, undergoing evaluation with upper GI endoscopy, and having available pathology results. We excluded patients receiving active anti-cancer treatments, those with active GI bleeding, and those using other anticoagulants such as warfarin and enoxaparin. All patients underwent the same standard gastroscopy procedure, performed by the same gastroenterologist. Two minutes before the start of the gastroscopy, patients were sedated with 1-3 mg of midazolam for sedation induction, followed by an infusion of 0.5 mg/kg of propofol as needed. The standard gastroscopy procedures were completed within 5-10 minutes. Biopsy procedures from any area of the gastric or duodenal mucosa were performed if the patient had no contraindications. The standard dose of Apixaban was 2x5 mg/day for treatment and 2x2.5 mg/day for prophylaxis or for patients with hepatic/liver failures. The standard dose for Rivaroxaban was 20 mg/day for treatment and 10-15 mg/day for prophylaxis or for patients with liver/kidney failures. The standard dose of dabigatran was 2x150 mg/day for treatment and 2x75 mg/day for prophylaxis or for patients with hepatic/liver failures. The standard dose of Edoxaban was 60 mg/day for treatment and 30 mg/day for prophylaxis or for patients with hepatic/liver failures.

The patients were divided into two groups according to their DOAC usage status. In all, 60 patients using DOACs for at least a month were defined as the DOAC group, whereas 65 patients with no history of DOAC use were defined as the control group. Then, the patients in the DOAC group were further divided into subgroups according to DOAC usage duration as "using DOAC<12 months" and "using DOACs ≥ 12 months," as demonstrated in Table 3. Further, patients in the DOAC group were divided into the subgroups Rivaroxaban group and Other DOACs group for analysis, as demonstrated in Table 4. Lastly, the patients were divided into the following subgroups: Rivaroxaban group, Apixaban group, and Other DOACs (combining dabigatran + edoxaban due to fewer numbers), as demonstrated in Table 5. Demographical, laboratory, and pathology results were obtained from hospital data processing systems and analyzed retrospectively. Blood test results were those performed on the day of upper GI endoscopy. A single pathologist evaluated upper GI pathology specimens, considering HP, AtG, AnG, upper GI ulcers, and IM. We found 60 eligible patients and included them in the study for the DOAC group between the years 2020 and 2023 from the hospital data processing system. The control group was selected randomly according to our inclusion criteria from the hospital data processing system, except for the close age and gender distribution with the DOAC group (patients with similar ages and gender distribution were selected for the control group, which means a probability sampling was performed).

Our study was approved by the Giresun Educational and Research Hospital Ethical Committee (acceptance date: September 11, 2023, meeting number: 170, approval number: 05). Informed consent was not applicable due to the retrospective design of our study. Our study was conducted in accordance with the Declaration of Helsinki. Personal information was kept confidential, and the privacy of participating research subjects was protected.

Sample size calculation

The G*Power 3.1.9.4 package program was used for sample size calculation. The sample size was calculated using independent sample T-test analysis to compare various parameters of patients using and not using DOACs. Cohen's d standardized effect size value is used in T-test analyses. Cohen's d value of 0.20, 0.50, and 0.80 is interpreted as small, medium, and high effect size, respectively [8]. Considering the effect size as 0.80, the N2/N1 ratio as 1.08, the probability of error as 0.05, and the power of the study as 0.99, it was seen that 125 people should be selected, of whom at least 60 used DOAC drugs and at least 65 did not use DOAC drugs.

Data analysis

The data were analyzed through the IBM SPSS Statistics for Windows, Version 26 (Released 2019; IBM Corp., Armonk, New York). Categorical data of the patients are given as numbers and percentages. In numerical data, only the mean and standard deviation are given for variables with a normal distribution, and for data that do not show a normal distribution, the median, minimum, and maximum values are given along with the mean and standard deviation. The compliance of the numerical variables of the patients with normal distribution was determined by looking at the Skewness and Kurtosis coefficients. The patients' ejection fraction (EF), alanine aminotransferase (ALT), aspartate aminotransferase (AST), lactate dehydrogenase (LDH), ferritin, B12, international normalized ratio (INR), C-reactive protein (CRP), white blood cell (WBC), and platelets (PLT) values did not show normal distribution. It has been observed that glomerular filtration rate (GFR), albumin, sodium (Na), chlor (Cl), potassium (K), calcium (Ca), hemoglobin (Hb), hematocrit (HCT), mean cellular volume (MCV), MCH (mean cellular hemoglobin), PLT, mean platelet volume (MPV), neutrophil, lymphocyte, and monocyte values comply with the rules of normal distribution. The reference value taken in the normal distribution is between ±1.5 [9]. The chi-square test was used to compare the DOAC drug use status and duration of DOAC drug use and the descriptive characteristics and pathology parameters of the patients. The independent sample T-test was used to compare laboratory parameters (GFR, albumin, Na, CL, K, Ca, Hb, HCT, MCV, MCH, PLT, MPV, neutrophil, lymphocyte, and monocyte) that showed normal distribution according to DOAC drug use status. Mann-Whitney U test was used to compare laboratory parameters (EF, ALT, AST, LDH, ferritin, B12, INR, CRP, WBC, and PLT) that showed normal distribution according to the DOAC drug use. The chi-square test was used to compare the pathology parameters of patients not using DOAC (control), patients using DOAC for more than 12 months, patients using rivaroxaban, and patients using other DOACs. In the entire study, significance levels were determined by taking into account the values of 0.05 and 0.01.

## Results

The patients' demographic data and gastric mucosa findings are compared in Table 1. There was no statistical difference in gender distribution between the groups. The median age for the DOAC group was approximately 75, whereas it was approximately 70 for the control group, and this difference was significant (p = 0.008). In the DOAC group, 46.7% of patients were on rivaroxaban, 35% on apixaban, 11.7% on edoxaban, and 6.7% on dabigatran. While six patients in the DOAC group did not have antral gastritis, all patients in the control group had antral gastritis, and this difference was statistically significant (p = 0.028). Although HP and IM were found at lower rates in the DOAC group, this difference was statistically insignificant. In addition, although upper GI ulcers and AtG were detected at a higher rate in the DOAC group, this difference was not statistically significant.

**Table 1:** The comparison of the control and DOAC groups regarding demographic data and pathology specimen results *p<0.05 **p<0.01 x^2^: chi-square test (categorical data), t: independent sample T-test, z: Mann-Whitney U test, mean±SD: mean ± standard deviation, Med: median, Min: minimum, Max: maximum, H. pylori: Helicobacter pylori, I. metaplasia: intestinal metaplasia, GI: gastrointestinal, S-B: stomach-bulbus co-existence

Parameters		Control Group (n = 65)				DOAC Group (n = 60)				p
		n		%		n		%		
Gender	Male	28		43.1		26		43.3		1.000
	Female	37		56.9		34		56.7		
H. pylori	No	45		69.2		44		73.3		0.758
	Yes	20		30.8		16		26.7		
Antral gastritis	No	0		0		6		10		0.028*
	Yes	65		100		54		90		
Atrophic gastritis	No	60		92.3		55		91.7		1.000
	Yes	5		7.7		5		8.3		
Upper GI ulcers	No	60		92.3		49		81.7		0.318
	Stomach	3		4.6		8		13.3		
	Bulbus	1		1.5		2		3.3		
	S-B	1		1.5		1		1.7		
I. metaplasia	No	52		80		55		91.7		0.109
	Yes	13		20		5		8.3		
DOAC type	Rivaroxaban	0		0		28		46.7		-
	Apixaban	0		0		21		35		
	Edoxaban	0		0		7		11.7		
	Dabigatran	0		0		4		6.7		
		Mean±SD	Med.	Min.	Max.	Mean±SD	Med.	Min.	Max.	
Age^t^		70.14±11.9	70	46	94	75.72±11.25	78.50	42	93	0.008**

The comparison of laboratory parameters between the groups is shown in Table 2. Glomerular filtration rate (GFR), albumin, potassium (K), calcium (Ca), hemoglobin (Hb), and hematocrit (HCT) were significantly lower (p = 0.001, 0.000, 0.008, 0.000, 0.000, 0.000, respectively), while vitamin B12, INR, and C-reactive protein (CRP) were significantly higher (p = 0.03, 0.000, 0.006, respectively) in the DOAC group. No other significant association was found regarding other lab parameters between the groups.

**Table 2:** The comparison of the laboratory parameters between the DOAC and control group *p<0.05 **p<0.01 t: independent sample T-test, z: Mann-Whitney U test, Mean±S.D.: mean ± standard deviation, Med: median, Min: minimum, Max: maximum, GFR: glomerular filtration rate, ALT: alanine aminotransferase, AST: aspartate aminotransferase, Na: sodium, Cl: chlor, K: potassium, Ca: calcium, LDH: lactate dehydrogenase, INR: international normalized ratio, CRP: C-reactive protein, WBC: white blood cell, HGB: hemoglobin, HT: hematocrit, MCV: mean corpuscular volume, MCH: mean corpuscular hemoglobin, PLT: platelets, MPV: mean platelet volume

Lab parameters	Control group (n = 65)				DOAC group (n = 60)				p
	Mean±SD	Med.	Min.	Max.	Mean±SD	Med.	Min.	Max.	
GFR^t ^(mL/min/1.73 m^2^)	79.31±21.05				65.31±23.75				0.001**
ALT^z^(u/L)	16.53±10.41				16.42±9.34	14.5	4	42	0.787
AST^z^(u/L)	20.53±9.72				22.75±9.90	20.5	7.23	51	0.124
Albumin^t^(g/L)	42.31±6.32				37.16±7.78				0.000**
Na^t^(mmol/L)	139.88±3.18				138.83±4.11				0.113
Cl^t^(mmol/L)	102.48±4.21				103.57±4.83				0.181
K^t^(mmol/L)	4.42±0.45				4.07±0.91				0.008**
Ca^t^(mg/dL)	9.37±0.64				8.83±0.86				0.000**
LDH^z^(u/L)	214.72±82.98	197.5	85	584	220.15±67.70	205	47	450	0.212
Ferritin^z^(ng/mL)	85.37±106.54	50.2	5.1	592.7	94.62±96.86	53.6	4.9	413	0.300
Vitamin B12^z^(pg/mL)	445.85±334.80	351	108	2000	621.00±483.84	457	169.3	2000	0.030*
INR^z^	1.09±0.24	1.03	0.87	2.21	1.22±0.23	1.12	0.91	1.84	0.000**
CRP^z^(mg/mL)	11.00±24.68	2	0	129.66	26.32±39.25	6	0.19	164.9	0.006**
WBC^t^ (10^9^/L)	7.53±3.53				7.52±4.02				0.530
HGB^t^(g/dL)	12.16±2.07				10.19±2.26				0.000**
HT^t^(%)	37.27±5.36				31.95±6.32				0.000**
MCV^t^ (µm^3^)	86.13±7.90				86.38±6.89				0.852
MCH^t^(pg/cell)	28.08±3.26				27.88±3.08				0.736
PLT^z^(10^9^/L)	247.25±89.10	226	41	568	237.75±91.33	226	84	662	0.557
MPV^t^(fL)	9.47±1.23				9.11±0.83				0.058
Neutrophils^t^(cells/mm^3^)	4155.65±1969.42				4731.58±2297.41				0.134
Lymphocytes^t^(cells/mm^3^)	1823.54±511.96				1682.64±661.99				0.187
Monocytes^t^(cells/mm^3^)	514.46±214.53				542.24±211.12				0.566

The comparison of the patients' descriptive characteristics and pathology parameters according to the duration of DOAC use was shown in Table 3, and no statistical difference was found between the groups regarding these parameters. The mean period of NOAC use was 24.07 months. The minimum use of DOAC was one month, whereas the maximum use was 88 months in the DOAC group. HP, AnG, AtG, upper GI ulcers, and IM percental were higher in the group using DOAC for 12 months or longer.

**Table 3:** The comparison of the patients using a DOAC for a year or more vs. for less than a year regarding age, gender, and pathology specimen results *p<0.05 **p<0.01 x^2^: chi-square test (categorical variables), t: independent sample T-test, Atroph: atrophic, Mean±SD: mean ± standard deviation, Med: median, Min: minimum, Max: maximum, H. pylori: Helicobacter pylori, I. metaplasia: intestinal metaplasia, GI: gastrointestinal, S-B: stomach-bulbus co-existence

Parameters		Using DOAC < 12 months (n = 29)						Using DOAC ≥ 12 months (n = 31)				p
		n			%			n		%		
Gender	Male	12			41.4			14		45.2		0.972
	Female	17			58.6			17		54.8		
H.pylori	No	22			75.9			22		71		0.892
	Yes	7			24.1			9		29		
Antral gastritis	No	4			13.8			2		6.5		0.605
	Yes	25			86.2			29		93.5		
Atroph. gastritis	No	27			93.1			28		90.3		1.000
	Yes	2			6.9			3		9.7		
Upper GI ulcers	No	27			93.1			22		71		0.142
	Stomach	2			6.9			6		19.4		
	Bulbus	0			0			2		6.5		
	S-B	0			0			1		3.2		
I. metaplasia	No	28			96.6			27		87.1		0.392
	Yes	1			3.4			4		12.9		
		Mean ±SD	Med.	Min.			Max.	Mean±SD	Med.	Min.	Max.	
Age^t^		75.03±10.81	79	42		89		76.35±11.80	77	45	93	0.654

In a subgroup analysis demonstrated in Table 4, we compared the pathology parameters of the patients using rivaroxaban vs. other DOACs. Patients using rivaroxaban significantly had less HP positivity than patients using other DOACs (p = 0.042). No other significant association was revealed regarding other pathology parameters.

**Table 4:** The comparison of patients using rivaroxaban vs. other DOACs regarding pathology specimen results *p<0.05 **p<0.01 x^2^: chi-square test (categorical data), Atroph: atrophic, H. pylori: Helicobacter pylori, I. metaplasia: intestinal metaplasia, GI: gastrointestinal, S-B: stomach-Bulbus co-existence

Parameters		Rivaroxaban Group (n = 28)		Other DOACs Group (n = 32)		p
		n	%	n	%	
H.pylori	No	24	85.7	20	62.5	0.042*
	Yes	4	14.3	12	37.5	
Antral gastritis	No	4	14.3	2	6.3	0.404
	Yes	24	85.7	30	93.8	
Atroph. gastritis	No	26	92.9	29	90.6	1.000
	Yes	2	7.1	3	9.4	
Upper GI ulcers	No	23	82.1	26	81.3	0.125
	Stomach	2	7.1	6	18.8	
	Bulbus	2	7.1	0	0	
	S-B	1	3.6	0	0	
I. Metaplasia	No	26	92.9	29	90.6	1.000
	Yes	2	7.1	3	9.4	

Finally, the last subgroup analysis compares the groups “Control,” “Rivaroxaban,” “Apixaban,” and “Other DOACs.” This analysis revealed that the Rivaroxaban group has the least antral gastritis (p = 0.024) and, although statistically insignificant, the least HP positivity. Other interesting but statistically insignificant results in this analysis include having the least AtG and IM for the Apixaban group and approximately similar rates of upper GI ulcers for all DOAC groups (Table 5). 

**Table 5:** The comparison of the control group, Rivaroxaban group, Apixaban group, and Other DOACs group with respect to pathology specimen results *p<0.05 **p<0.01 x^2^: chi-square test (categorical data), Atroph: atrophic, H. pylori: Helicobacter pylori, I. metaplasia: intestinal metaplasia, GI: gastrointestinal, S-B: stomach-bulbus co-existence

Parameters		Control Group (n = 65)		Rivaroxaban Group (n = 28)		Apixaban Group (n = 21)		Other DOACs Group (n = 11)		p
		n	%	n	%	n	%	n	%	
H.pylori	No	45	69.2	24	85.7	14	66.7	6	54.5	0.195
	Yes	20	30.8	4	14.3	7	33.3	5	45.5	
Antral gastritis	No	0	0	4	14.3	1	4.8	1	9.1	0.024*
	Yes	65	100	24	85.7	20	95.2	10	90.9	
Atroph. gastritis	No	60	92.3	26	92.9	20	95.2	9	81.8	0.677
	Yes	5	7.7	2	7.1	1	4.8	2	18.2	
Upper GI ulcers	No	60	92.3	23	82.1	17	81	9	81.8	0.311
	Stomach	3	4.6	2	7.1	4	19	2	18.2	
	Bulbus	1	1.5	2	7.1	0	0	0	0	
	S-B	1	1.5	1	3.6	0	0	0	0	
I. Metaplasia	Yok	52	80	26	92.9	20	95.2	9	81.8	0.213
	Var	13	20	2	7.1	1	4.8	2	18.2	

## Discussion

In this study, we tried to reveal whether DOACs have local effects on gastric mucosa. We compared patients with and without DOAC use and performed subgroup analyses, presenting exciting results. First, AtG and IM were less frequently seen in the DOAC group. The second interesting result was the higher prevalence of upper GI ulcers in the DOAC group. The third one was the worsening of gastric mucosa findings in patients using DOAC for a year or more compared to ones using DOAC for less than a year. The final significant result was the lower prevalence of HP and AnG in patients using rivaroxaban compared to ones using other DOACs and the control group. These findings are unique and loadstar to future trials since no similar study is presented in the literature as far as we know. Potential pathophysiological mechanisms and etiologies underlying these results will be discussed as follows. 

Textbooks, reviews, and randomized controlled trials in the literature have mainly focused on the anticoagulant effect and bleeding side effects of DOACs so far [3,5-7]. However, there could be some other implications on the different parts of the body since these drugs are systemic. A nationwide study conducted with French National Healthcare databases in 2018 revealed associations of DOAC use with the initiation of antidepressants, anti-constipation, antiemetic medications, and presentation of acute liver injury, kidney diseases, and hospitalized bleeding [10]. This study showed that DOACs may have systemic side effects without proving it with mucosal or pathological specimen changes. Another case reported hemorrhagic gastritis following the onset of Dabigatran therapy in an 85-year-old patient with renal insufficiency [11]. Despite these rare articles, the literature did not report any other studies regarding the local and systemic side effects of these brand-new popular drugs. Our study is the first study reporting the local impacts of DOACs on the upper GI system with proof of pathology specimens. The lower prevalence of AnG and IM in the DOAC group was consistent with our hypothesis, indicating that DOACs might have healing effects on the gastric mucosa. At this point, Rivaroxaban comes to the forefront among other DOACs, as Table 4 and 5 reveals its significant association with reduced AnG and HP prevalence. The underlying mechanism for this reduced prevalence should be studied, and the association should be confirmed with randomized controlled trials(RCTs). For example, a contradictory study suggested that Rivaroxaban was associated with higher GI bleeding rates than apixaban and dabigatran [12]. However, this study investigated the effects of DOACs only in terms of GI bleeding risk rather than all gastric mucosa effects. 

Recent studies showed that anticoagulant agents may have antitumoral effects since coagulation proteins have been suggested to increase tumor growth and angiogenesis [13,14]. Several in vitro studies reported that Dabigatran and Apixaban might have antitumoral activity, especially when combined with chemotherapeutic agents [15-17]. Concordantly, our results revealed that dyspeptic patients using DOACs, particularly rivaroxaban and apixaban, had markedly reduced intestinal metaplasia in their gastric biopsy results (Tables 1, 5). This result is crucial since it might have clinical effects such as choosing the suitable anticoagulant for patients with cancer and combining anti-cancer treatments with proper DOACs. However, these results, along with previous in vitro studies suggesting similar results, should be verified by RCTs in vivo before impacting the daily routines of clinicians.

Other concerns preventing us from being sure about the beneficial effects of DOACs were the higher prevalence of upper GI ulcers in the DOAC group and the worse pathological results in the group using DOAC for a year and more. Our hypothesis for these controversial results is that the possible local anticoagulation and increased blood circulation in microvessels caused by DOACs might have beneficial effects on normal gastric mucosa, such as preventing antral gastritis and intestinal metaplasia, etc. However, prolonged use of DOACs might worsen some local preexisting lesions in the upper GI system and transform them into ulcers by damaging mucosa and submucosa of the gastric tissue caused by the same local anticoagulant effect. It may be possible that these negative effects of DOACs on gastric lesions might be limited with the addition of a proton pump inhibitor to the therapy. Our second hypothesis for the increased ulcer prevalence in the DOAC group is that the increased microcirculation in the gastric mucosa caused by DOACs may somehow lead to stimulation of parietal cells, hence increasing hydrochloric acid (HCl) secretion, which is a known risk factor for upper GI ulcers [18]. It is unknown whether the effects of DOACs on gastric mucosa are transitory or weakened with prolonged use. All these hypotheses should be enlightened with the results derived from future RCTs on this issue. 

HP infection is considered one of the most critical risk factors for upper GI bleeding side effects for patients using DOACs [3,19]. A retrospective observational study of 260 HP-positive patients revealed that adding anti-HP treatment in the DOAC group reduces upper GI bleeding risk, severity of bleeding, surgical intervention, and upper GI bleeding-associated mortality rates [20]. A recent population-based study comparing the upper GI bleeding risks of newly started DOACs and warfarin in HP-eradicated patients vs. HP-negative patients suggested a reduced risk of upper GI bleeding in new users of DOACs [21]. These two studies showed us the importance of HP status in determining anticoagulant therapy and the risk of upper GI bleeding. Unfortunately, there are no RCTs conducted with large patient populations investigating the association of anti-HP treatment with anticoagulant treatments in the literature. Our results revealed a significantly reduced prevalence of HP in the rivaroxaban group compared with the other DOACs group. This result suggests that rivaroxaban should be the drug of choice among all DOACs for patients with concomitant HP infection. This unique relationship has never been demonstrated in the literature so far and therefore needs to be studied in RCTs. Other analyses regarding HP in our study include a slightly reduced rate of HP negativity in the DOAC group and a similar rate of HP positivity in new and old DOAC users (Tables 1, Table 3). 

Lastly, comparing lab parameters between the DOAC and control group revealed negative results such as lower GFR, K, Ca, HB, HCT, and higher CRP for the DOAC group (Table 2). These results are unsurprising for us since patients using DOACs are expected to have more comorbidities such as atrial fibrillation, cerebrovascular diseases, deep venous thromboembolism, and pulmonary thromboembolism [22]. However, what is surprising here is the generally favorable outcomes of the gastric mucosa specimens in the DOAC group while having worse lab parameters. This association doubles the novelty of this study’s results. Unfortunately, there are limitations in our study. First, the retrospective design and some missing data in the patients’ files, such as pictures of the pathology specimens and gastroscopy procedures, the history of other drug use, HP positivity, pathology results, and comorbidities of the patients, weakened the analyses. Second, the study consisted of a small patient group. Third, fewer patients were in the Dabigatran and Edoxaban groups; therefore, we could not add these groups separately to the analyses. Finally, there was a small but statistically significant age difference between the groups. Nevertheless, as the authors, we think that the novelty of our results deserves interest and lights the way for future RCTs to evaluate this untouched part of the literature. 

## Conclusions

In conclusion, our study revealed that dyspeptic patients on DOACs were less likely to have antral gastritis and intestinal metaplasia when compared to the control group. Rivaroxaban is the DOAC that comes into prominence among others with its lower HP and antral gastritis positivity. Higher upper GI ulcers and weakening effects with prolonged usage are controversial results regarding the beneficial effects of DOACs on gastric mucosa. Rivaroxaban might be chosen while determining a proper DOAC for dyspeptic patients with cancer and HP positivity. More studies are required to prove the effects of DOACs on gastric mucosa. 
